# Deciphering the Role of CD14 in *Helicobacter Pylori*-associated Gastritis and Gastric Cancer: Combing Bioinformatics Analysis and Experiments

**DOI:** 10.7150/jca.106847

**Published:** 2025-03-03

**Authors:** Xuefei Yang, Jiaqi Zhang, Ping Wang, Fengyun Wang, Xudong Tang

**Affiliations:** 1 Department of Gastroenterology, Peking University Traditional Chinese Medicine Clinical Medical School (Xiyuan), Beijing, China.; 2 Institute of Digestive Diseases, Xiyuan Hospital of China Academy of Chinese Medical Sciences, Beijing, China.

**Keywords:** CD14, gastric cancer, *Helicobacter pylori*-associated gastritis, immunology, immunotherapy, biomarkers

## Abstract

**Background:** Gastric cancer (GC) is the third leading cause of cancer-related death and is associated with high mortality and morbidity. *Helicobacter pylori* (HP) infection is the most important cause of GC. We aimed to identify the core genes of HP caused GC and further elucidate the underlying mechanisms.

**Methods:** GC and HP associated gastritis (HPAG) gene expression data were sourced from Gene Expression Omnibus. Key genes affecting GC prognosis were identified using Cytoscape software. Patient groups were formed based on key gene expression, and the immune analyses were performed with R. MNU, derived from nitrite by HP, was given to GC mice (240ppm) for histology and fluorescence assays. For in vitro experiments, cells received MNU (20 μM) stimulation for 24 hours.

**Results:** CD14 was the only key gene identified. A total of 412 GC patients were divided into CD14-high and CD14-low groups. The two groups showed significant differences in immune cell populations and immune checkpoints. In particular, there was a notable increase in M2 macrophages in GC patients with high CD14 expression (*P* <0.001). GC Patients with high CD14 expression exhibited a more pronounced immune response than those with low CD14 expression, and elevated CD14 expression positively correlated with the efficacy of CTLA4 therapy (*P* <0.05). These results indicated that CD14 expression was strongly correlated with the GC immune response. A noticeable increase in CD14 levels was observed in MNU-induced GC animals, cell models, and GC patients. In addition, the number of M2 macrophages was increased in MNU-induced GC mice.

**Conclusion:** Reducing CD14 expression may increase the survival rate of GC patients through the modulation of immune responses. The complex mechanism of CD14's influence on prognosis deserves further investigation.

## Introduction

The gastric is a unique saccular organ. Tumor growth in the gastric cannot cause noticeable symptoms in the early stages, leading to a poor prognosis of gastric cancer (GC)^1^. GC is the fifth most common cancer and the third most common cause of cancer death worldwide^2^. Understanding the molecular characteristics of GC and detecting molecular markers for early warning help ensure that patients receive timely intervention. Although significant progress has been made in novel therapies for GC, surgical and endoscopic resection remain the primary treatments for GC. This is because the pathophysiology of GC remains unclear, and the treatment strategies such as immune checkpoint blockade require more investigation^3^. Therefore, evaluating new novel biomarkers related to immunotherapy is urgent.

The inclusion of inflammation in the revised “Hallmarks of Cancer” highlights the crucial role of inflammation in the progression of cancer^4^. GC is one of the few cancers directly linked to infectious agents. According to a well-acknowledged GC-progression model, the progression of GC follows the model: “gastritis-intestinal metaplasia-low grade neoplasia-high grade neoplasia-GC”^5^. *Helicobacter pylori*-associated gastritis (HPAG) is the earliest step of GC progression^6^, ^7^. Common genes between HPAG and GC may be the predominant factor that results in the genetic susceptibility of the host. *Helicobacter pylori* can persist in the gastric for decades, damaging the mucosa, changing the release of gastric hormones, and disrupting gastric physiology^8^. Previous reports have provided important insights into how *helicobacter pylori* target different proteins to influence the inflammatory response in the gastric mucosa and lead to GC^8^-^11^. Although significant efforts have been conducted to determine the relationship between HPAG and GC, the jury is still inconclusive.

Here, we provide an account of new biomarkers and molecular targets that enhance understanding of the relationship between HPAG and GC. Investigations into GC have faced constraints due to the contentious nature of animal model usage. Although mouse models have had limited success in accurately representing GC, several models have effectively captured the most important characteristics of GC progression^12^. A compound derived from dietary nitrite by the conversion action of* helicobacter pylori*, an N-nitroso species named N-Methyl-N-nitrosourea (MNU), triggers somatic mutations in epithelial cells, leading to gastric premalignancy^13^. In summary, MNU is the predominant carcinogenic derivative of *helicobacter pylori*, and its carcinogenicity model can effectively mimic the carcinogenic effects of the bacterium. Specifically, the carcinogenic potential of MNU to the Fore- stomach tumor and glandular stomach tumor is significantly greater compared to other MNU analogs and healthy control groups^14^. Therefore, we employed MNU for both *in vitro* and *in vivo* experiments to reveal the principal mechanisms that *helicobacter pylori* induced GC.

## Materials and Methods

### Identification of differentially expressed genes

**Figure 1:**
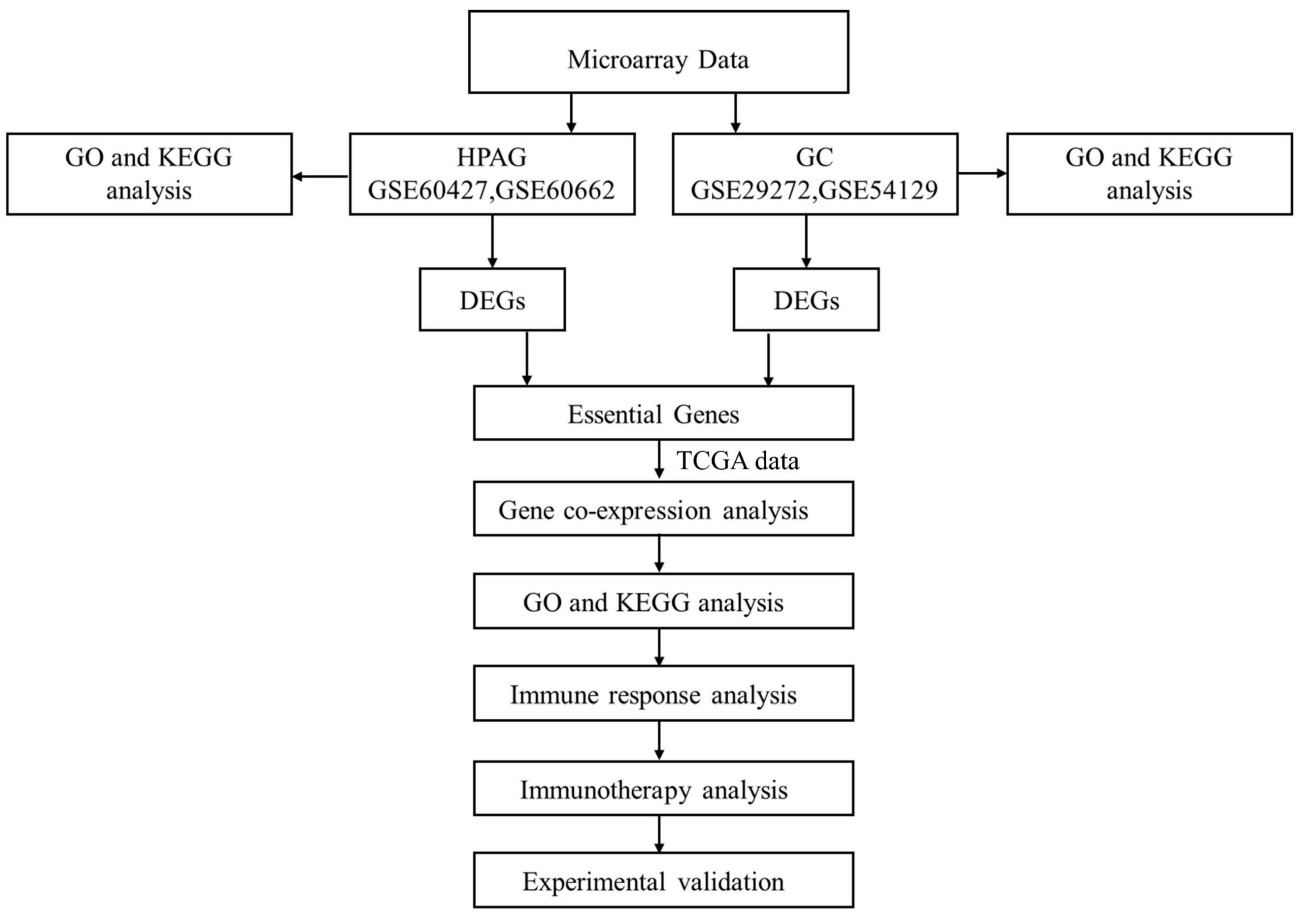
Analysis flow chart.

Four datasets were collected from the Gene Expression Omnibus (GEO) database. A flow chart is shown in Figure 1. GEO2R was used to screen the differentially expressed genes (DEGs) between the patients and the control groups^15^. DEGs of GSE29272 and GSE54129 were defined as the GC group. DEGs of GSE60427 and GSE60662 were defined as HPAG groups. Benjamini and Hochberg's false discovery rate method was applied to correct the *P*^16^. In our research, a *P* < 0.05 and |Log fold change|>1.0 were set as the identification thresholds for DEGs^17^.

### DEG enrichment analysis and hub gene identification

DEGs of the GC group and the HPAG group were used for further investigation. Functional enrichment and interaction networks were identified by the Gene Oncology (GO) analysis through Funrich^18^. The Kyoto Encyclopedia of Genes and Genomes (KEGG) pathway was determined using DAVID^19^. The KEGG pathway data were visualized via Origin software. The protein-protein interaction (PPI) network of DEGs was analyzed by the STRING network^20^. We employed the maximal clique centrality method of the cytoHubba app in Cytoscape software (version 3.8.0) to find the top 100 genes in the two groups^21^. All works were repeated twice.

### Prognosis analysis

The expression of hub genes was performed using Gene Expression Profiling Interactive Analysis^22^. The *P-*cutoff was 0.01, and we used log2 (TPM + 1) for the log scale x. The prognosis analysis was performed through the Kaplan-Meier plotter, which contains 1065 gastric samples from the GEO and the Cancer Genome Atlas^23^-^25^. Overall survival, progression-free survival, and post-progression survival analyses of hub genes were assessed. Hub genes with high prognostic value were defined as key genes.

### Enrichment analysis based on key gene-associated genes

The Cancer Genome Atlas was used to collect data on GC patients^26^, ^27^. The detailed clinic parameters of enrolled patients can be downloaded from The Cancer Genome Atlas. According to the expression of key genes, the GC patients were divided into a high-expression group (HEG) and a low-expression group (LEG). DEGs of HEG and LEG were gathered for further GO and KEGG analysis to elucidate the role of the key gene in the progression of GC, and all the results were visualized through the package include “Pheatmap”, “Enrichr”, and “Circlize”.

### Immune response analysis

Immune response analysis of key genes was carried out by R programming language. The immune scores of the HEG and the LEG groups were compared. Levels of immune cells between the HEG and LEG samples were visualized by the “ESTIMATE” package. R programming language was employed to analyze the relevance of key genes and different immune cells with a *P*-cutoff of 0.05. Besides, we valued the correlation between the immune checkpoints and key genes by the “Ggplot2” package. Besides, we carried out an immunotherapy analysis of key genes by the “Limma” package with the data downloaded from The Cancer Immunome Database.

### Experiment verification

12 mice (C57/6J, 20-25g) were divided into two groups (the GC group and the normal group). 240 ppm of N-nitroso-N-methylurea (MNU) was given to the GC group at 10 weeks while the normal group was fed with normal drinking water (Figure 11A). After another 20 weeks of normal feeding, the mice were sacrificed for further investigation. The weight of the body and spleen were evaluated. The gastric corpus of mice was fixed in 10% buffered formalin for H&E staining, immunohistochemistry, immunofluorescence, and Alcian Blue/Phosphoric Acid Schiff Staining (AB-PAS). The slides were viewed by Case Viewer under 100 × and 200 × microscope.

Human gastric mucosal epithelial cells were analyzed by STR gene detection. 10% fetal bovine serum (BI, Australia) was added to DMEM (Gibco, USA) for cell incubation under 5% CO_2_ at 37 °C. When the cells reached 70-80% confluence, the MNU group was treated with MNU (20 µM) for 24 h, while the normal group received the same volume of PBS. Both groups of cells were harvested for analysis of mRNA according to the RNA sequence (Table 1). The experiments were repeated three times. Besides, protein expression in GC patients was revealed by immunohistochemistry.

## Results

### Immune response is the shared mechanism of GC and HPAG

**Figure 2:**
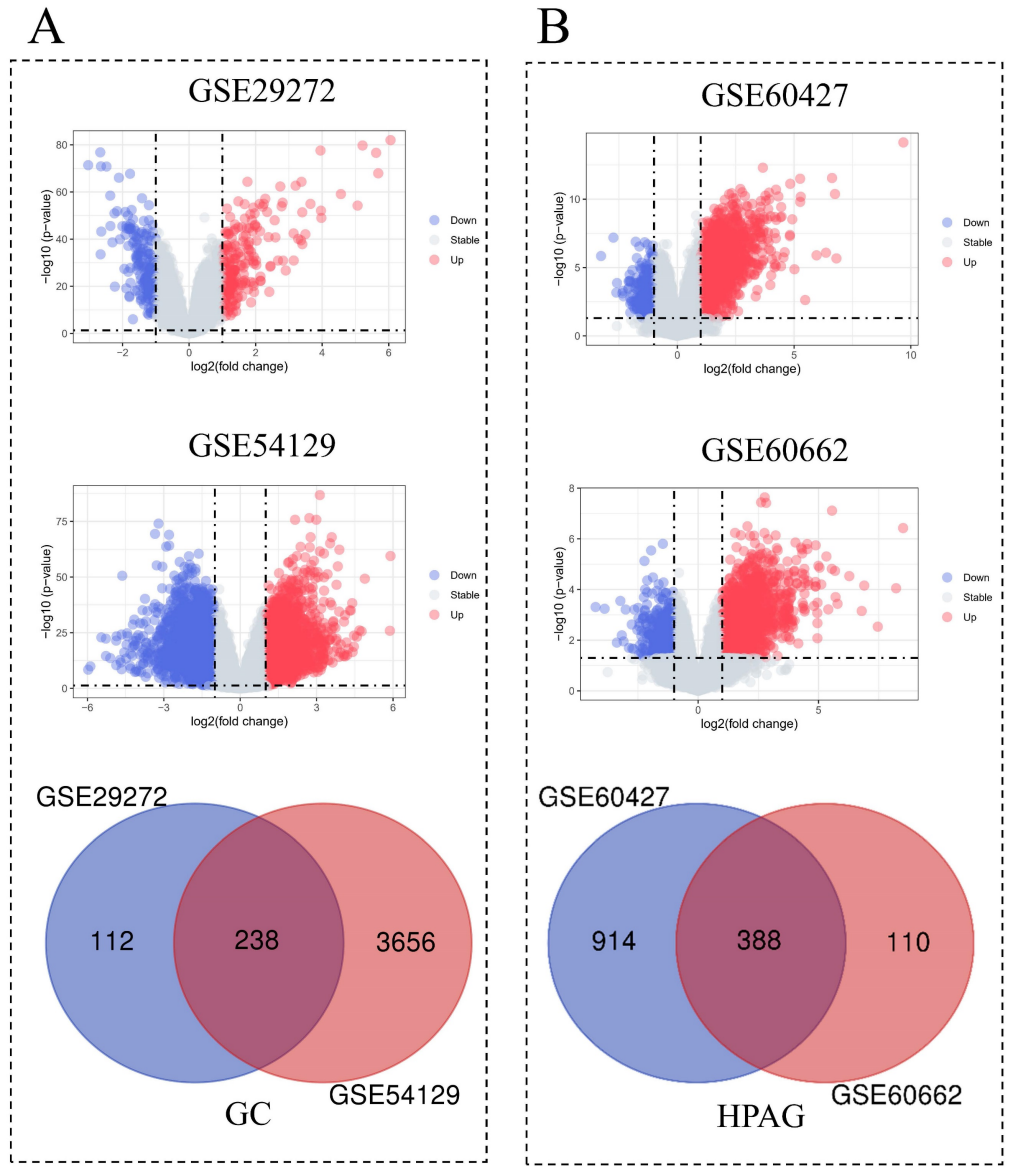
Identification of differentially expressed genes (DEGs) of *helicobacter pylori*-associated gastritis (HPAG) and gastric cancer (GC). A: Analysis of HPAG, including volcano plots of DEGs for datasets GSE60427 and GSE60662 and Venn diagrams of DEGs in GC; B: Analysis of GC, including volcano plots of DEGs for datasets GSE29272 and GSE54129 and Venn diagrams of DEGs in HPAG.

**Figure 3:**
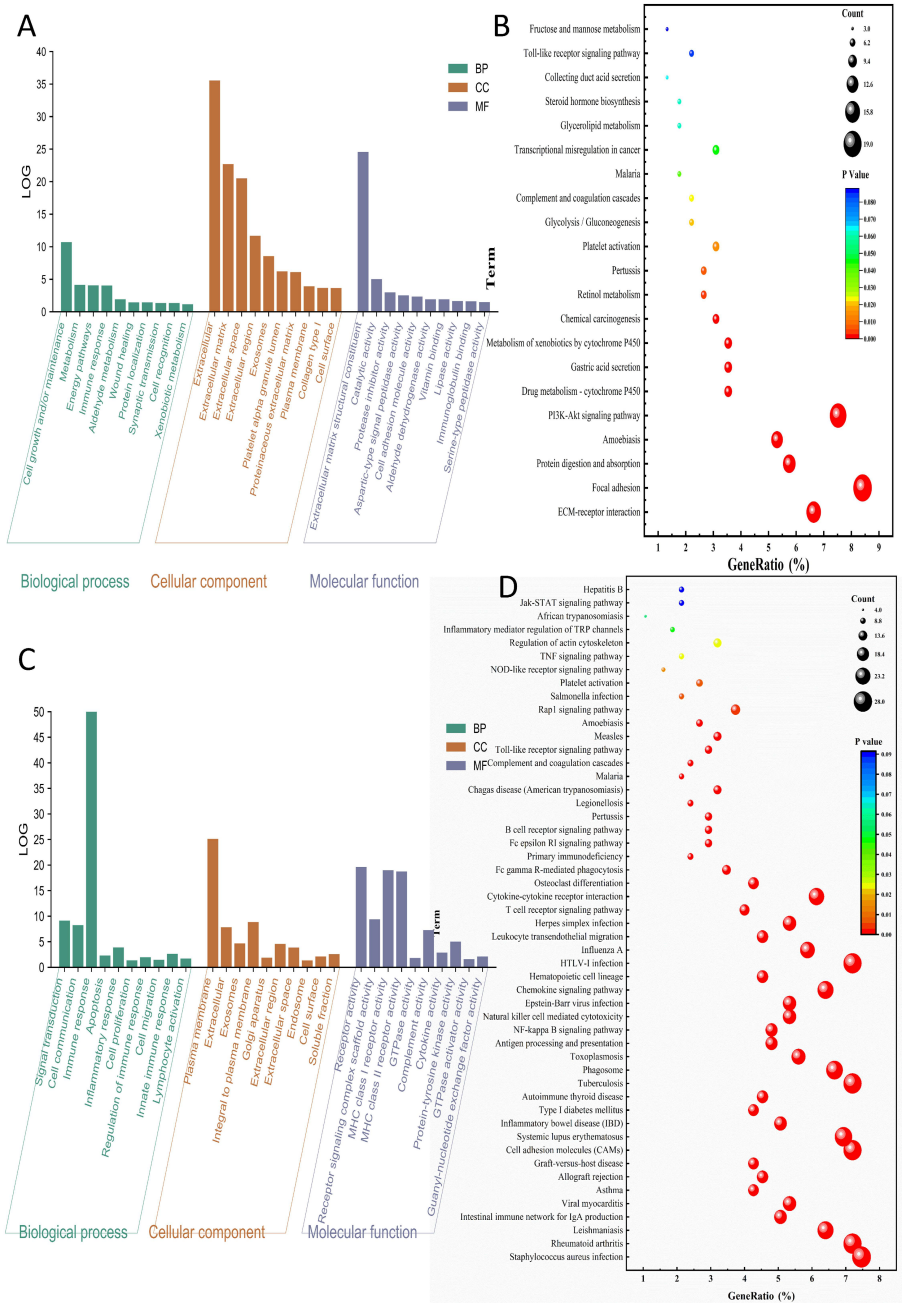
Enrichment analysis of DEGs in the GC group (A-B) and HPAG group (C-D). The top 10 results are shown. A, C: Biological process analysis, cellular component analysis, and molecular function analysis; B, D: KEGG pathway analysis.

Among the two GC datasets, there are 134 tumor samples and 134 adjacent normal samples in GSE29272, while GSE54129 includes 111 tumor tissues and 21 normal tissues. In the HPAG dataset, GSE60427 contains 16 samples of gastritis and 8 normal samples, GSE60662 includes 8 HPAG samples and 4 controls. 238 DEGs of GC and 388 DEGs of HPAG were screened for subsequent analysis (Figure 2). The GO analysis was performed to reveal the biological change of GC and HPAG. Biological process enrichment of the GC group indicated that cell growth and/or maintenance (*P* < 0.001) was the leading function (Figure 3). The results of cellular component and molecular function in the GC group proved that extracellular (*P* < 0.001) and extracellular matrix structural constituents (*P* < 0.001) play an important role. In the HPAG group, the top molecular function is the immune response (*P* < 0.001), the most critical enriched cellular component is the plasma membrane (*P* < 0.001), and receptor activity (*P* < 0.001) takes the top place in the analysis of molecular function. Both groups' immune response was enriched in the biological process (Figure 3A-C).

KEGG pathway analysis indicated that the DEGs in the GC group were enriched mainly in the ECM-receptor interaction (count=15, gene ratio=6.63%, *P* < 0.001). The HPAG group exhibited Staphylococcus aureus infection (count=28, gene ratio=7.46%, *P* < 0.001). Immune activity-associated pathways, such as the Toll-like receptor signaling pathway, were enriched in both the GC and HPAG groups (Figure 3B, D). The immune response disorder is a common mechanism of GC and HPAG.

### CD14 was the only key gene with a high prognostic value

**Figure 4:**
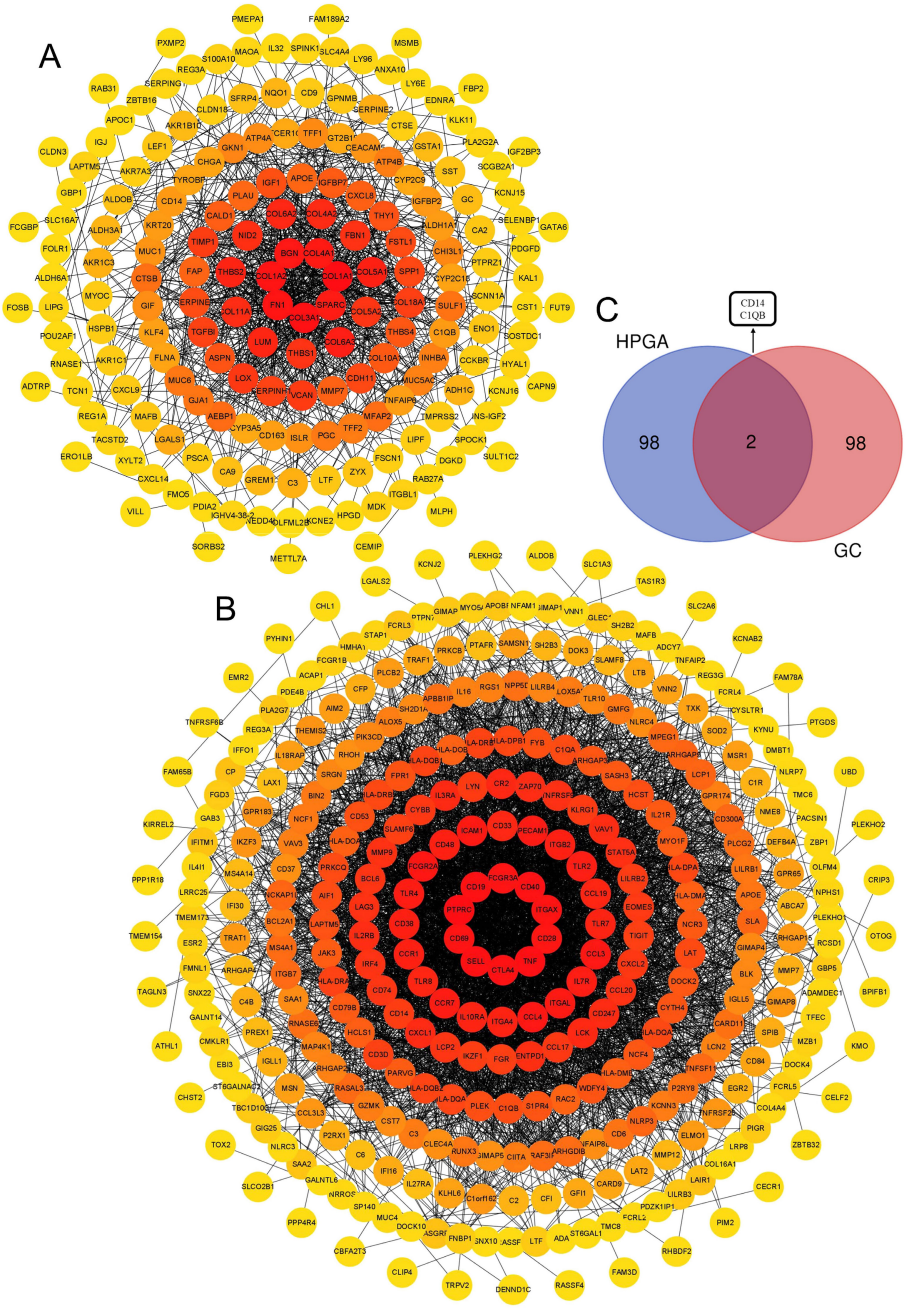
A, B: Protein-protein interaction (PPI) of the DEGs of the GC group (A) and HPAG group (B). The DEGs in the center of the circle mean higher scores. C, Venn diagram of the top 100 genes in the 2 groups. CD14 and C1QB are the hub genes. The red genes showed higher scores and were gathered in the figure's heart.

There were 184 nodes and 926 pairs of interactions between them in the GC group's PPI network (Figure 4A). A total of 330 nodes and 3867 pairs of interactions between them were observed in the PPI network of the HPAG group (Figure 4B). The genes were chosen by an interaction score higher than 0.4 in the STRING platform. The two hub genes (CD14 and C1QB) were found to co-exist in the GC and HPAG groups among the top 100 genes of the two groups (Figure 4C).

**Figure 5:**
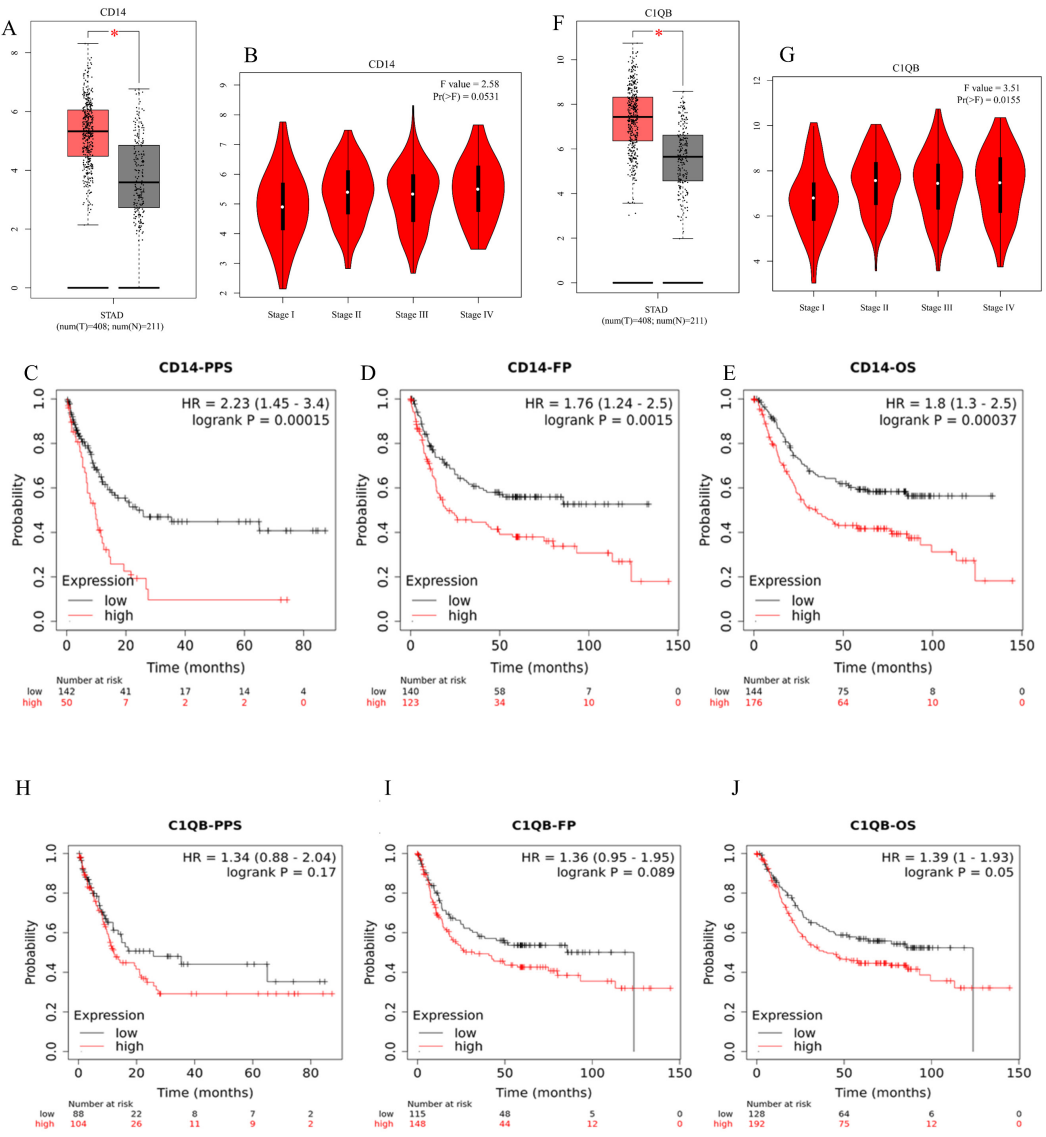
Clinical analyses of CD14 and C1QB. A, Expression analysis of CD14. B, The relationship between CD14 and GC stage. C-E: Post Progression Survival, progression-free survival, and overall survival analyses of CD14 in GC. F, The expression analysis of C1QB; G, The relationship of C1QB and GC stages; H-J: Post Progression Survival; Progress Free Survival, and overall survival, analyses of C1QB in GC. (A, F: tumor color: red, *: *P* < 0.05).

The expression levels of C1QB and CD14 in GC were significantly higher than those in the normal group (*P* < 0.05). In the meanwhile, the expression of CD14 showed no significant association with the cancer stage of GC patients, while C1QB expression was significantly related to the GC stage (Figure 5B and G, *P* < 0.05). According to the Kaplan-Meier plotter, CD14 mRNA expression was associated with GC in overall survival, progression-free survival, and post post-progression survival (Figure 5C-E, *P* < 0.05). However, C1QB showed no relationship in the survival analysis of GC (Figure 5H-J, *P* > 0.05). CD14 was screened as the only key gene in our research.

### CD14-related genes mainly participate in the immune response of GC

**Figure 6:**
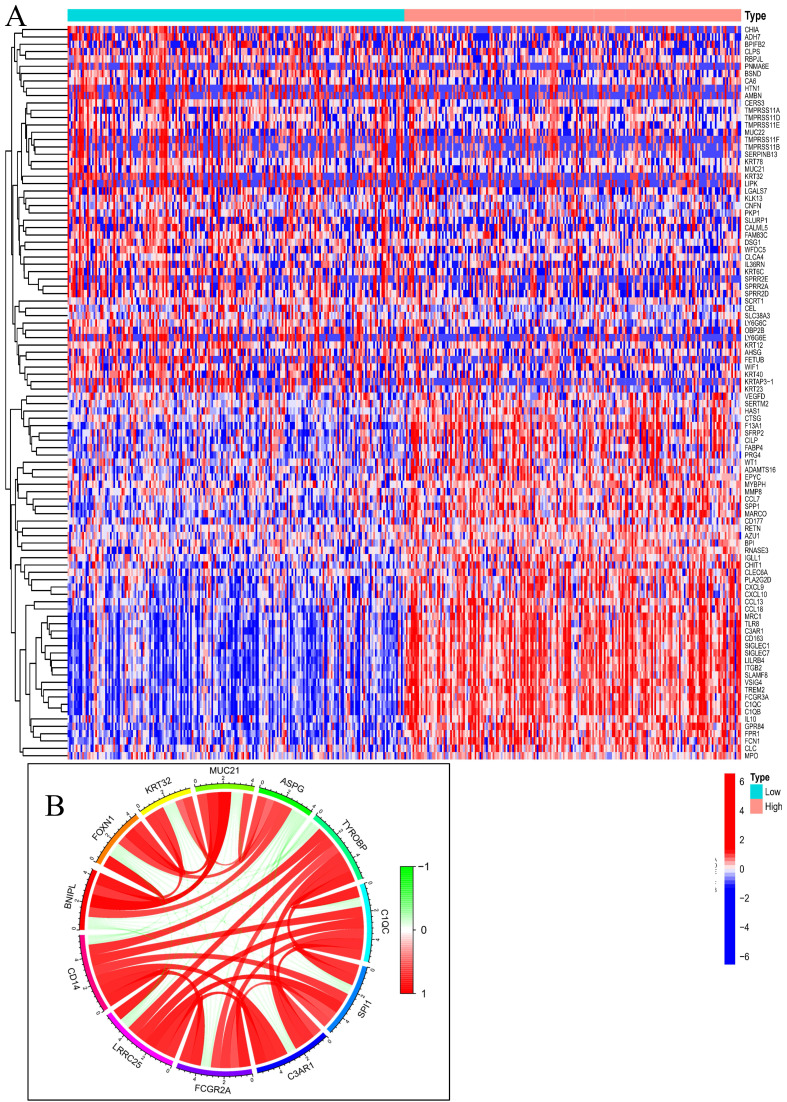
Gene correlation analysis of CD14 in GC. A, Differentially expressed genes in the CD14-high expression group and CD14-low expression group; B, Top 10 CD14-related genes; red indicates a positive correlation, and green indicates a negative correlation.

To determine the relationship between GC and CD14, 412 GC patients were downloaded from the Cancer Genome Atlas for further enrichment analysis. Compared the CD14 HEG to the CD14 LEG groups, a total of 18,650 genes were analyzed, and 318 genes had a |Cor|>0.6 and a *P*-value<0.001. We found that immune-related genes such as IL10, CD163, CXCL9, and CXCL10 were increased in the HEG group (Figure 6A). The top 10 CD14-related genes were LRRC25, FCGR2A, C3AR1, SPI1, C1QC, TYROBP, ASPG, MUC21, KRT32, FOXN1, and BNIPL (Figure 6B).

**Figure 7:**
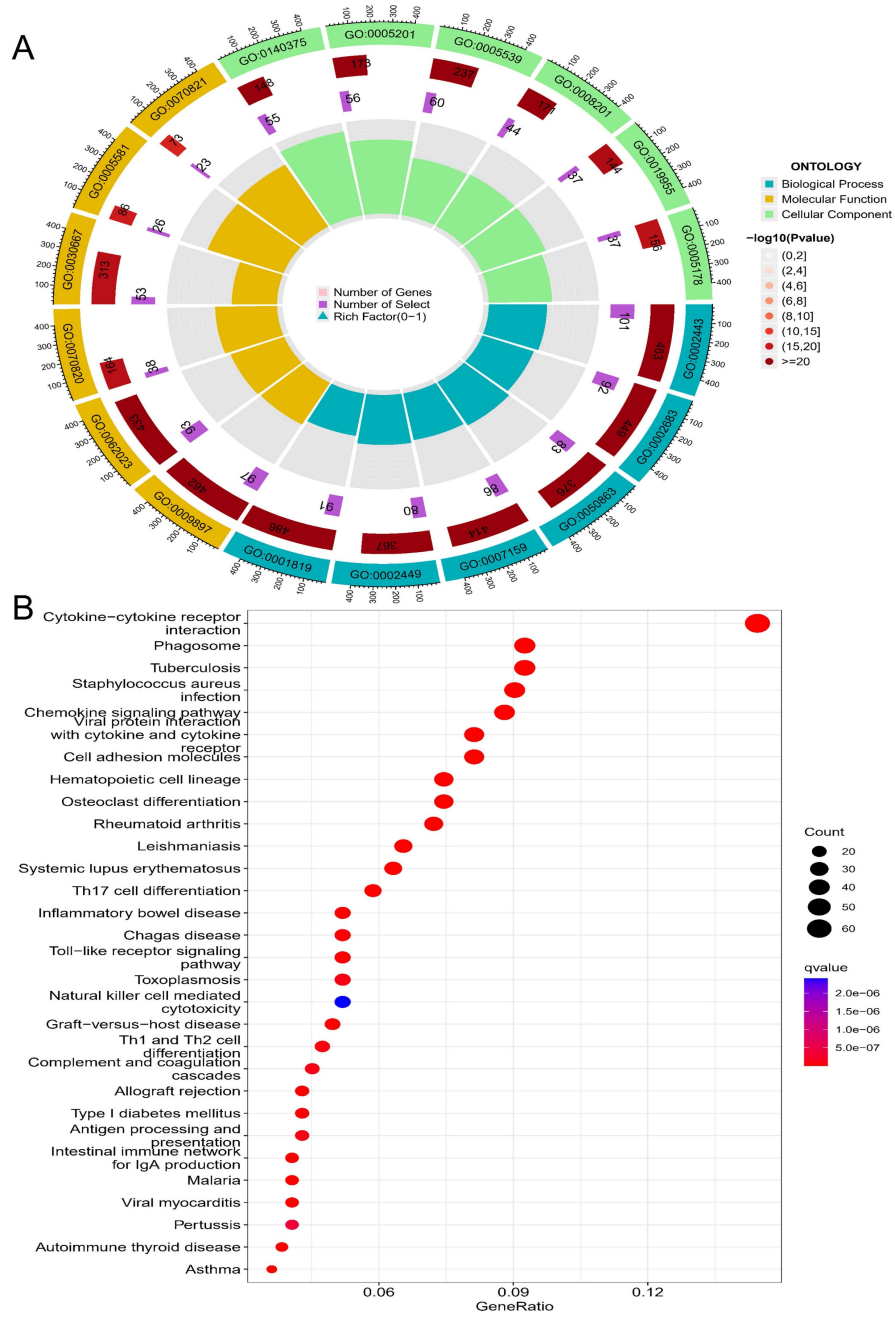
Enrichment analysis of CD14-related genes. A, top 6 of each GO analysis; B, top 30 of KEGG pathways.

GO analysis revealed that CD14-related genes were involved in immune-related cell functions such as leukocyte-mediated immunity, negative regulation of immune system process, regulation of T-cell activation, and immune receptor activity (Table 2, Figure 7A). KEGG pathway enrichment analysis suggested that the CD14-related genes play a significant role in cytokine-cytokine receptor interaction, Toll-like receptor signaling pathway, viral protein interaction with cytokine and cytokine receptor, and the Th17 cell differentiation (Figure 7B).

### CD14 expression related to immune cells and immunotherapy of GC patients

**Figure 8:**
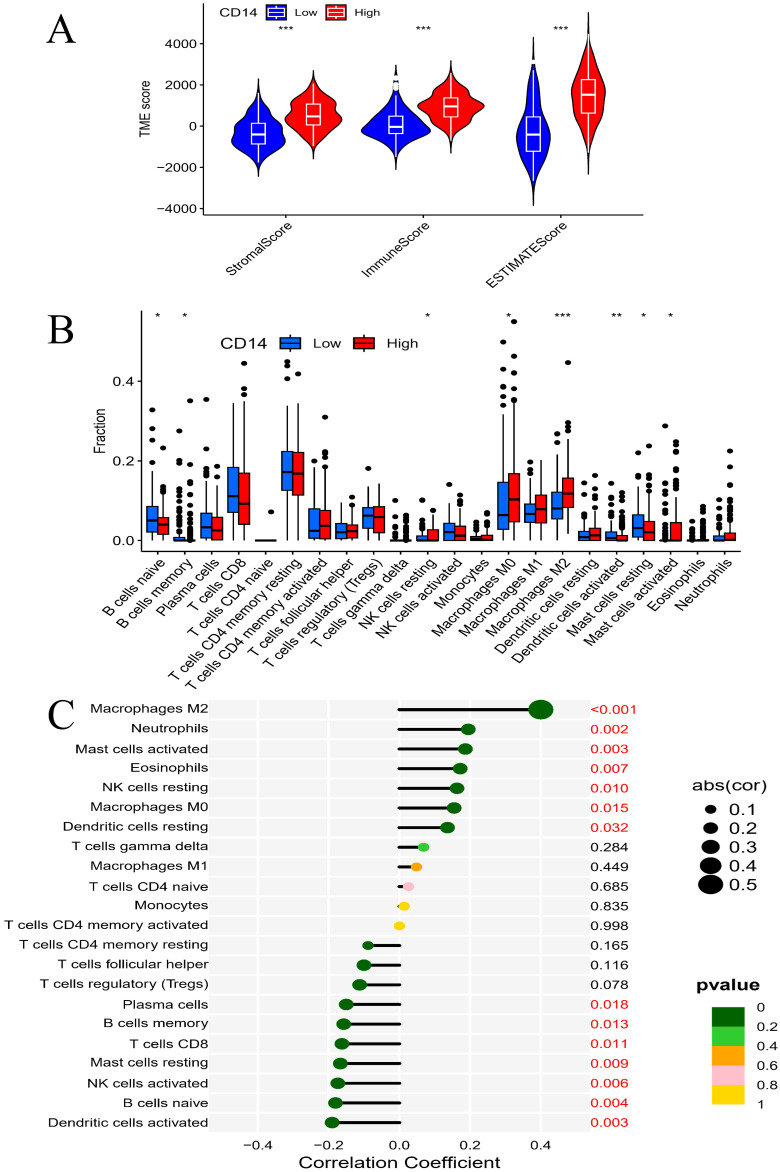
Correlation analysis of CD14 and immune cells. A, Immnue scores of different CD14 expression groups; B, Immnue cells of different CD14 expression groups; C, Relation analysis of CD14 and different kinds of immune cells (*: *P* < 0.05; **: *P* < 0.01; ***: *P* < 0.001. red number in C shows *P* < 0.05).

**Figure 9:**
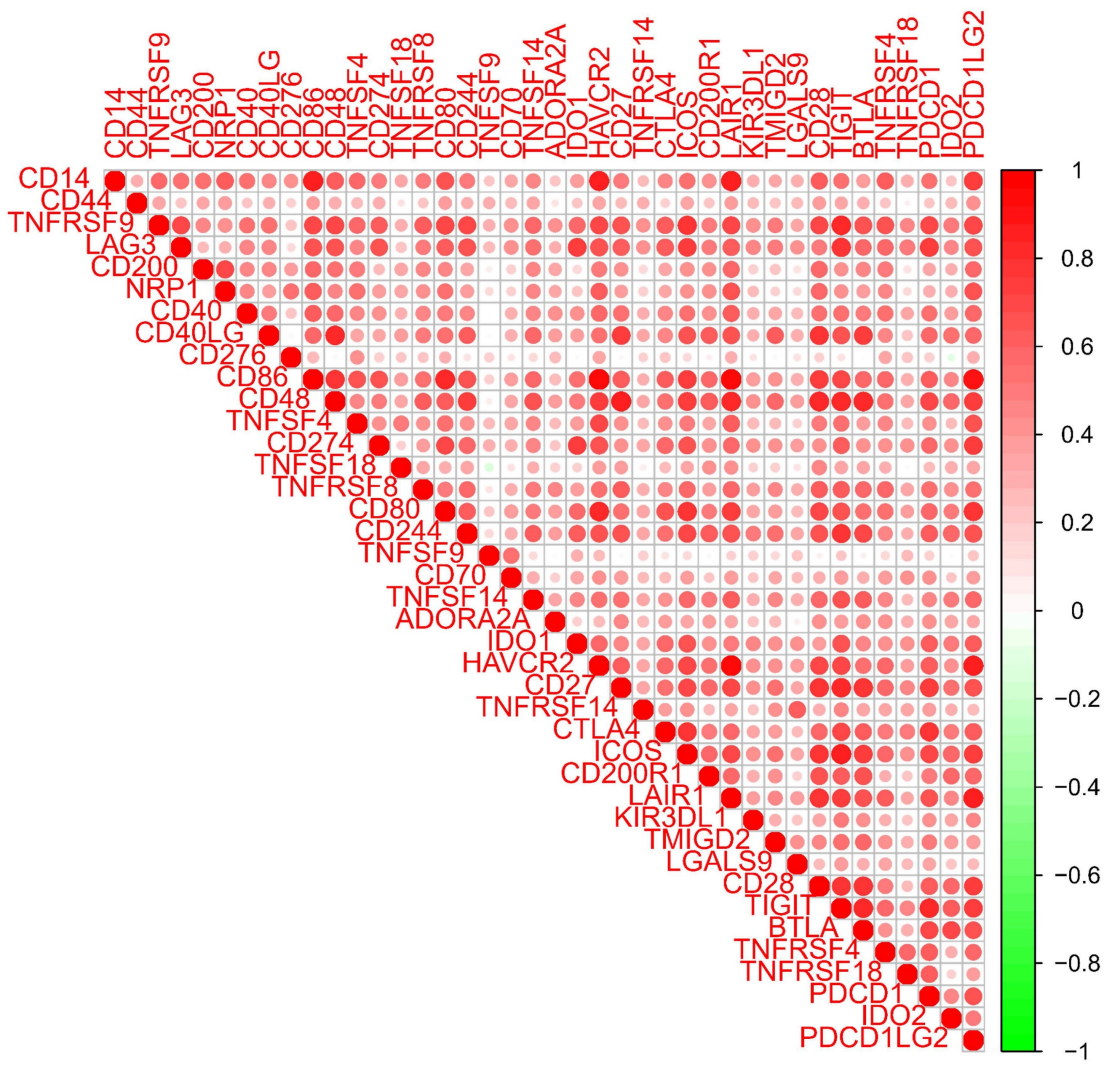
Relationship between CD14 and immune checkpoints. Red indicates a positive correlation, and green indicates a negative correlation.

The LEG group had a higher stromal score, immune score, and estimate score (all *P* < 0.001, Figure 8A), which indicated that higher expression of CD14 was associated with higher stromal and immune cell content in GC patients. Differential analysis of immune cells revealed that CD14 played a vital role in GC patients' immune response by regulating the expression of immune cells. Naive B cells, memory B cells, activated dendritic cells, and resting mast cells were significantly decreased in the HEG group, while resting NK cells, M0 macrophages, M2 macrophages, and activated mast cells increased dramatically in the HEG (all *P* <0.05, Figure 8B). The correlation analysis of CD14 and immune cells indicated that 14 kinds of immune cells strongly correlated with CD14 expression. Specifically, M2 macrophages were increased in the HEG and had a CD14-related result of Cor=0.4, P= 6.56E-11 (Figure 8). CD14 was significantly associated with all 39 immune checkpoints of GC, among which CD86, CD48, LAIR1, PDCD1LG2, and HAVCR2 had a Cor >0.8 (Figure 9, all *P* <0.001).

**Figure 10:**
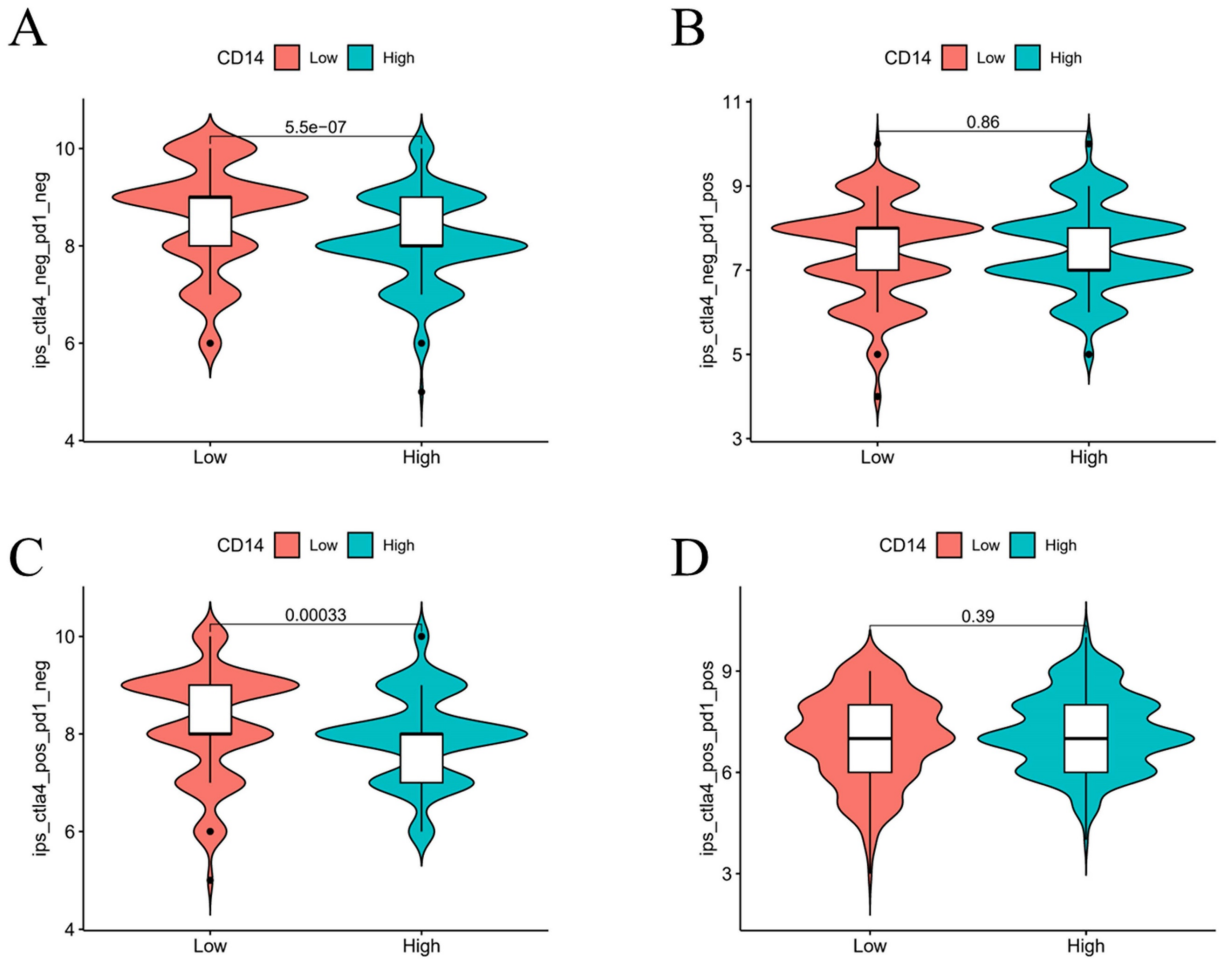
Influence of CD14 expression on the efficacy of immunotherapy.

The LEG had a higher score in both the CTLA4-negative & PD-1-negative group (*P* < 0.05, Figure 10A) and the CTLA4-positive & PD-1-negative group, indicating that the LEG had a better immunotherapy effect when patients received anti-CTLA4 therapy. The combination of anti-CTLA4, anti-PD-1 therapy, and anti-PD-1 therapy alone did not show significant differences between the two groups. The expression of CD14 may influence the effect of immunotherapy.

### CD14 increased in cells, animals, and patients of GC

**Figure 11:**
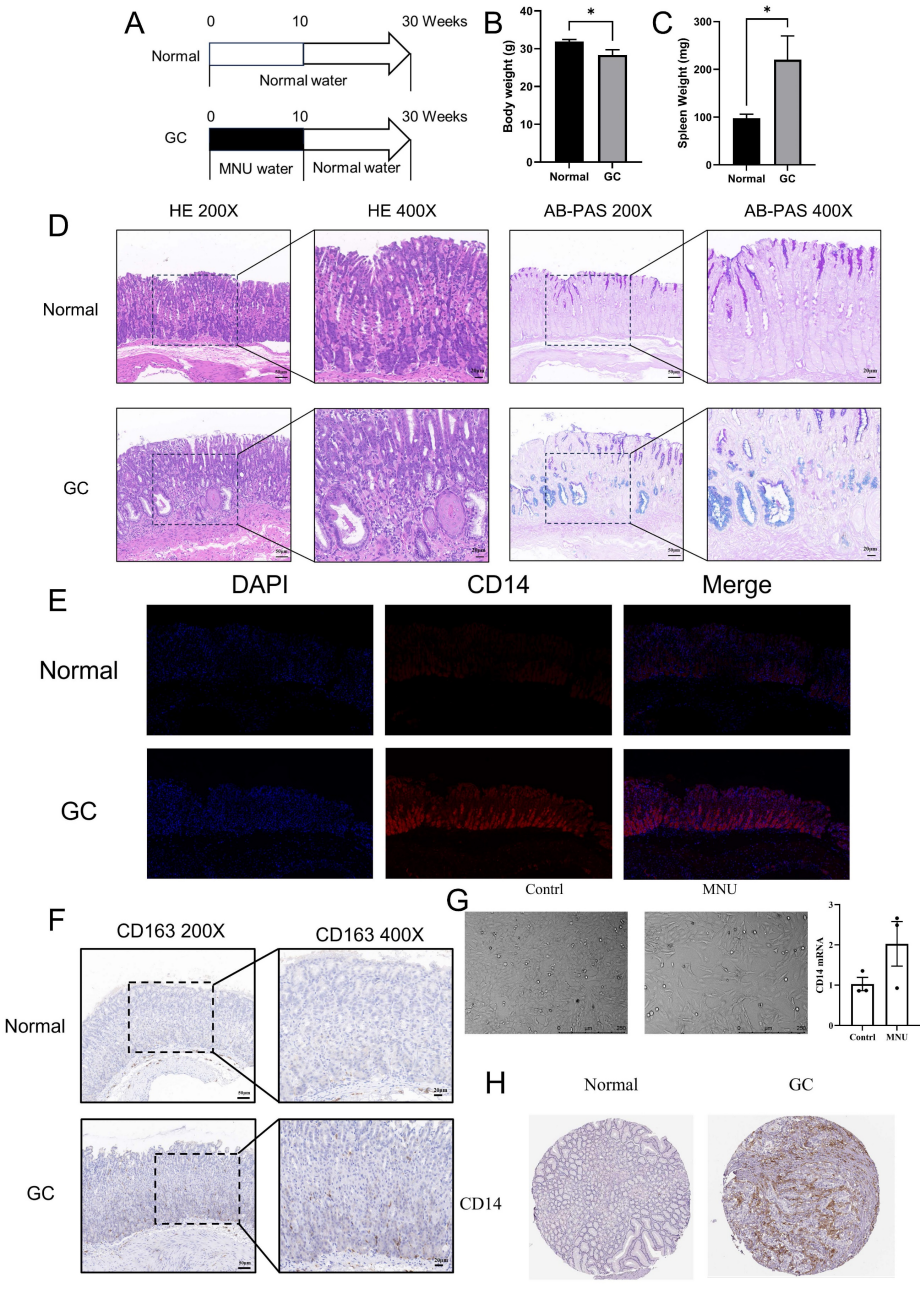
Expression analysis of CD14. A, GC animal model formation; B Body weight of GC mice; C, Spleen weight of GC mice; D HE and AB-PAS stain of GC mice; E, immunofluorescence stain of CD14 expression in GC mice; F, CD163 expression in GC mice; G Cell form and CD14 mRNA change after MNU stimulation; H, CD14 expression in GC patients. (*: *P* < 0.05).

MNU, an N-nitroso compound, was converted from dietary nitrite by *helicobacter pylori*. Previous reports found that MNU can cause somatic mutations in epithelial cells and induce gastric premalignancy^13^. In the MNU group (treated with 240 ppm MNU), the body weight of mice decreased significantly along with significantly increased spleen weight (Figure 11B-C, both *P* <0.05). HE staining showed that the gastric mucosal epithelial structure was disturbed, accompanied by obvious blue staining of AB-PAS, indicating the mutation of cells in the MNU group (Figure 11D). In the immunofluorescence results, CD14 expression increased significantly in the MNU group (Figure 11E). Besides, the M2 macrophage marker CD163 increased in the GC group along with the migration of macrophages from the base of the lineal body upward (Figure 11F), which was verified by cell experiments. After stimulation with MNU for 24 h, morphological changes occurred in Human gastric mucosal epithelial cells. CD14 mRNA was elevated in the MNU group when compared with the normal group (Figure 11G). Further analysis of the CD14 expression in GC patients found that the protein expression of CD14 was increased (Figure 11H).

## Discussion

*Helicobacter pylori* infection is the most common cause of GC, affecting nearly half of the global population^8^, ^28^, ^29^. The developmental mechanism from HPAG to GC is still unknown. Over the past few decades, various studies have been conducted to identify genes, such as m6A-associated genes, that may play an essential role in the development of GC^30^, ^31^. To identify persistently altered genes that influence the host's genetic susceptibility in GC and HPAG, we novelly proposed an analysis using different datasets and methods to identify the hub genes with potential diagnostic and prognostic values^32^, ^33^. CD14 and C1QB were identified using the GO and KEGG analysis (Figure 4C), and we further conducted prognosis analyses of these two genes in intestinal-type gastric cancer, as HPAG usually progresses into intestinal-type gastric cancer. CD14 was recognized for the first time as a key gene related to the prognosis of GC patients (Figure 5).

CD14 is a bacterial lipopolysaccharide receptor and a pattern recognition receptor, known for enhancing innate immune responses. It plays a vital role in transferring bacterial lipopolysaccharide to Toll-like receptor 4, triggering downstream signals such as NF-κB^34^. However, CD14 plays a controversial role in cancer regulation. Activated CD14 can increase tumor growth in bladder cancer^35^, while CD14 activation decreases cancer cell viability and induces apoptosis in adrenocortical carcinoma^36^, ^37^. In an analysis of HP-induced GC, CD14 is significantly related to both the progression and regression of the disease, indicating its role in dictating the trajectory of HP-induced GC^38^. Our findings revealed that elevated CD14 expression is associated with an unfavorable prognosis in GC, and it plays a role in the progression of HP-induced GC (Figure 5). Therefore, we contributed to a deeper understanding of the role of CD14 in HP-induced GC.

CD14-related genes were primarily involved in the immune response (Figure 6 and Figure 7). High CD14 expression was associated with higher immune scores (Figure 8A). There are two forms of CD14: membrane-bound CD14 and soluble CD14. Membrane-bound CD14 is highly expressed in myeloid lineage cells, including monocytes, DCs, macrophages, and microglia. Soluble CD14 exists in body fluids such as the serum, conferring an immune response to cells that do not express CD14^36^. As CD14 is expressed by monocytes/macrophages, it is logical that the group with high CD14 expression had significantly elevated macrophage expression. Notably, M2 macrophage expression was dramatically elevated in the HEG compared to the LEG (Figure 8B). This finding is consistent with previous studies indicating that M2 macrophage can promote inflammatory responses and have tumorigenic functions^39^. Our results also shown that CD14 expression can significantly influence the levels of naive B cells and memory B cells (Figure 8B, C), which may be due to CD14 regulating the immune network by influencing immune checkpoint in GC (Figure 9). Alternatively, this may result from the increased levels of soluble CD14.

Immune checkpoint blockade has been recognized as a useful therapy^3^. For example, PD-1 is one of the most common checkpoint blockade therapies used in many cancers including GC^40^, ^41^. However, some researchers have found that PD-1 may lead to immune tolerance and exhibit poor therapeutic effects^42^, ^43^. Therefore, it is crucial to determine the immune network of GC and improve the tumor response to immunotherapy. In our study, CD14 was identified as an essential DEG that governs GC prognosis and the immune response. Low CD14 expression was predicted to have a higher response only to anti-CTLA4 immunotherapy (Figure 10A-D). Complementing the effectiveness of anti-PD-1 immunotherapy, combinations of immune checkpoint blockade, including anti-CTLA4 and anti-PD1, may be more effective.

In summary, we conducted cell and animal experiments to verify CD14 expression. This work will contribute to a deeper understanding of immune response in GC. However, there are some limitations in our study. Essential factors such as age were missing due to the complexity of the datasets. While CD14 is known as a negatively correlated gene in GC, its expression may be involved in more complex changes during the progression of GC. The role of different forms of CD14 has not been analyzed. Further investigation into CD14 and more evidence on the biological basis of GC and HPAG are needed. Additionally, while we have identified CD14 as a key player in the immunopathogenesis of GC, the precise molecular mechanisms by which CD14 influences tumor progression and immune evasion require further elucidation. Future studies should aim to dissect the complex interactions between CD14 and other immune regulators, and how these interactions shape the tumor microenvironment. Looking ahead, the translational potential of our findings warrants exploration. The role of CD14 in mediating the efficacy of immunotherapies, such as CTLA4 blockade, suggests that CD14 expression levels could serve as a predictive biomarker for patient response to treatment. Clinical trials incorporating CD14 as a stratification factor are warranted to assess whether personalized treatment approaches based on CD14 status can enhance therapeutic efficacy.

## Figures and Tables

**Table 1:** Probe, Primer, and Product (bp) for qPCR.

Gene	Upstream primer	Downstream primer	Product (*bp*)
CD14	GGACTTGCACTTTCCAGCTT	CCCAGTCCAGGATTGTCAGA	203
GAPDH	CACCCACTCCTCCACCTTTGA	TCTCTCTTCCTCTTGTGCTCTTGC	188

**Table 2:** GO analysis results.

ONTOLOGY	ID	Description	Gene Ratio	*p* Value	Count
Biological process	GO:0002443	leukocyte mediated immunity	101/822	6.18E-43	101
Biological process	GO:0002683	negative regulation of immune system process	92/822	9.10E-37	92
Biological process	GO:0050863	regulation of T cell activation	83/822	1.04E-35	83
Biological process	GO:0007159	leukocyte cell-cell adhesion	86/822	7.36E-35	86
Biological process	GO:0002449	lymphocyte mediated immunity	80/822	5.09E-34	80
Biological process	GO:0001819	positive regulation of cytokine production	91/822	3.89E-33	91
Cellular component	GO:0009897	external side of plasma membrane	97/875	3.72E-39	97
Cellular component	GO:0062023	collagen-containing extracellular matrix	93/875	2.11E-38	93
Cellular component	GO:0070820	tertiary granule	38/875	1.78E-17	38
Cellular component	GO:0030667	secretory granule membrane	53/875	2.22E-17	53
Cellular component	GO:0005581	collagen trimer	26/875	2.22E-15	26
Cellular component	GO:0070821	tertiary granule membrane	23/875	3.39E-14	23
Molecular function	GO:0140375	immune receptor activity	55/837	8.01E-36	55
Molecular function	GO:0005201	extracellular matrix structural constituent	56/837	9.20E-33	56
Molecular function	GO:0005539	glycosaminoglycan binding	60/837	1.83E-28	60
Molecular function	GO:0008201	heparin binding	44/837	1.77E-21	44
Molecular function	GO:0019955	cytokine binding	37/837	3.05E-18	37
Molecular function	GO:0005178	integrin binding	37/837	5.36E-17	37

## Abbreviations

GC: gastric cancer
DEGs: differentially expressed genes
GO: gene oncology
HPAG: *helicobacter pylori*-associated gastritis
KEGG: Kyoto encyclopedia of genes and genomes
GEO: Gene Expression Omnibus
MNU: N-nitroso-N-methylurea
PPI: protein‒protein interaction
HEG: high-expression group
LEG: low-expression group
